# The Association between Diet and Hepatocellular Carcinoma: A Systematic Review

**DOI:** 10.3390/nu13010172

**Published:** 2021-01-08

**Authors:** Elena S. George, Surbhi Sood, Anna Broughton, Georgia Cogan, Megan Hickey, Wai San Chan, Sonal Sudan, Amanda J. Nicoll

**Affiliations:** 1 Institute for Physical Activity and Nutrition, School of Exercise and Nutrition Sciences, Deakin University, Geelong 3217, Australia; surbhi.sood@deakin.edu.au (S.S.); apbrough@deakin.edu.au (A.B.); gcogan@deakin.edu.au (G.C.); mvh@deakin.edu.au (M.H.); wschan@deakin.edu.au (W.S.C.); sudanson@deakin.edu.au (S.S.); 2 Department of Gastroenterology, Eastern Health, Box Hill 3128, Australia; Amanda.Nicoll@easternhealth.org.au

**Keywords:** hepatocellular carcinoma, primary liver cancer, liver cancer, diet, dietary patterns, nutrition

## Abstract

Globally, liver cancer is the sixth most common cause of cancer mortality, with hepatocellular carcinoma (HCC) being the most common type of primary liver cancer. Emerging evidence states that diet is recognised as a potential lifestyle-related risk factor for the development of HCC. The aim of this systematic review is to determine whether there is an association between diet and the development of HCC. Using the PRISMA guidelines, three databases (MEDLINE Complete, CINAHL and Embase) were systematically searched, and studies published until July 2020 were included. Thirty observational studies were selected. The protocol was registered with PROSPERO (CRD42019135240). Higher adherence to the Mediterranean dietary pattern, Alternative Healthy Eating Index-2010, the Urban Prudent Dietary Pattern, the Traditional Cantonese Dietary Pattern, intake of vegetables, wholegrains, fish, poultry, coffee, macronutrients such as monounsaturated fats and micronutrients such as vitamin E, vitamin B9, β-carotene, manganese and potassium were associated with a reduced risk of HCC. The results suggest a potential role of diet in the development of HCC. Further quantitative research needs to be undertaken within a range of populations to investigate diet and the relationship with HCC risk.

## 1. Introduction 

Liver cancer is the seventh leading cause of cancer-related mortality in Australia, and the incidence continues to increase [1,2,3]. Hepatocellular carcinoma (HCC) is the most common type of primary liver cancer (PLC), accounting for approximately 90% of all cases [4,5,6]. HCC most commonly occurs in patients with liver cirrhosis [7,8] particularly in the setting of chronic hepatitis B virus (HBV) and hepatitis C virus (HCV) infections, dietary aflatoxin exposure, excessive alcohol consumption, tobacco smoking, and metabolic-associated fatty liver disease (MAFLD) [1,9]. Liver cirrhosis confers a very high risk of developing HCC, with between 5% and 30% of cirrhotic patients being diagnosed with HCC within the first five years [4]. Other identified risk factors for HCC include obesity, diabetes and MAFLD [10]. Diet has been recognised as a potential lifestyle-related risk factor for the development of HCC [11,12,13,14,15,16]. A healthy diet may play a preventative role in the development of some cancers, while a poor diet has been shown to increase cancer risk [11,14]. However, there is no clear consensus of what a protective diet is composed of for HCC risk [11].

Epidemiological evidence on the association of fruit and vegetable intake with gastrointestinal cancers suggests a protective role of a plant-based diet [11,12] Similarly, a positive association between increased processed meat consumption and breast cancer has been described [13]. However, little research has investigated the effect of diet on the development of HCC. The research that does exist is inconsistent and focuses on specific nutrients rather than food groups or dietary patterns. Thus, we identified the importance of focusing on dietary food groups (including macro- and micronutrients) and patterns in HCC prevention. Several reviews in the past have attempted to elucidate the potential association between nutrition and HCC [2,17,18,19]. However, there are no recent and or systematic literature reviews using the Preferred Reporting Items for Systematic Reviews and Meta-analysis (PRISMA) guidelines performed to date. Thus, the aim of the present systematic review is to determine whether there is an association between diet and dietary patterns, and the development of HCC.

## 2. Materials and Methods 

### 2.1. Search Terms and Strategy

In accordance with the PRISMA guidelines, a systematic search of MEDLINE Complete, CINAHL and EMBASE databases was conducted to source articles published from inception to July 2020. The protocol was registered with PROSPERO (International Prospective Register of Systematic Reviews), registration number CRD42019135240, prior to commencement. The research question was structured in accordance with the PICOS (Population, Intervention, Comparator, Outcome and Study Design) criteria (Table 1). The initial database searches were conducted by AB, GC and SS. Search terms used for the study selection were ‘diet*’ OR ‘diet* pattern*’ OR ‘diet* intervention’ OR ‘nutrient*’ OR ‘diet* intake’ OR ‘nutrition’ OR ‘calorie restrict*’ OR ‘hypercaloric’ OR ‘food*’ AND ‘hepatocellular carcinoma’ OR ‘HCC’ OR ‘liver cancer’ OR ‘liver tumo*’ OR ‘hepatoma’.

### 2.2. Eligibility Criteria 

Studies were included if they were written in the English language, included HCC as an outcome (either HCC prevalence at baseline or assessed risk at the conclusion of the study) and had a primary focus on associations between diet and HCC. Observational studies were included to determine dietary intake and the risk of HCC development. Articles were excluded if they were: not human studies, abstract only, review articles, focused on specific compounds such as aflatoxin or supplements rather than overall dietary patterns or food groups, focused on other mediating variables such as serum biomarkers, investigated other liver diseases such as MAFLD and cirrhosis or investigated broader lifestyle patterns such as physical activity or weight management and did not report on specific dietary patterns or components. 

### 2.3. Study Selection Process

**Figure 1:**
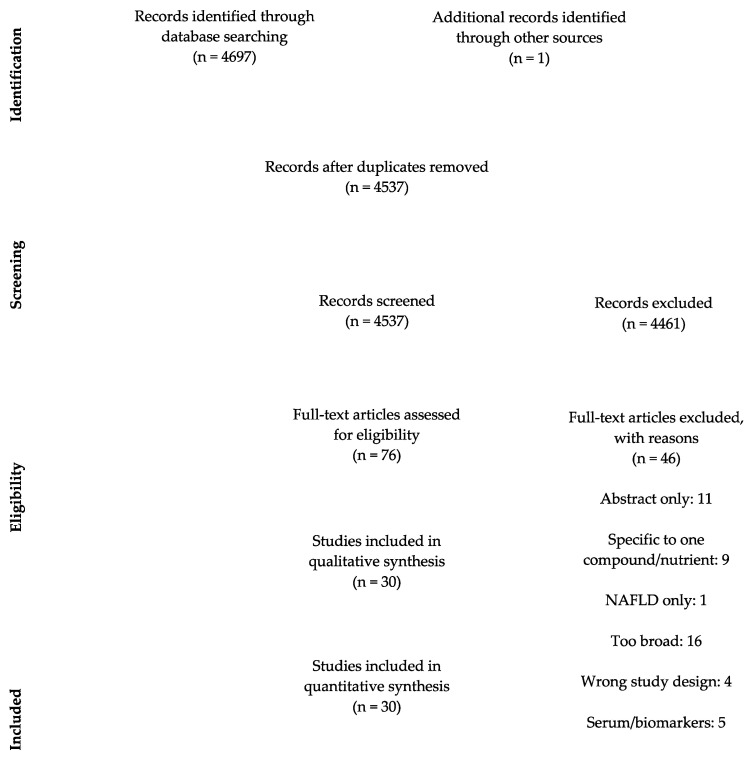
PRISMA flowchart of study selection process. NAFLD: Non-alcoholic fatty liver disease.

Abstract and title screening was conducted for all articles found in the initial search, and duplicates and articles which did not meet the eligibility criteria were excluded. Full-text screening was conducted by two independent reviewers. Conflicts were resolved through consensus. All articles accepted from the full-text screen were included in the systematic review. All inclusions are outlined in the PRISMA flowchart in Figure 1.

### 2.4. Data Extraction

The information extracted from 30 studies included the following: setting, duration, study design, population characteristics (age, sex, BMI, comorbidities), dietary patterns assessed in the study, dietary data assessment methods and outcomes.

### 2.5. Quality Assessment and Risk of Bias

The Academy of Nutrition and Dietetics Evidence Analysis Library Quality Criteria Checklist was used to assess overall quality and risk of bias. The questions are shown in Table 2. The risk of bias was conducted in duplicate independently for each study. Key questions assessed the studies overall relevance and validity. More specifically, these included whether the selection of participants was free from bias, whether groups were comparable, whether method of handling withdrawals/dropouts was described, whether blinding was used to prevent bias, whether interventions were described in detail with outcomes clearly defined, whether the statistical analysis was appropriate, whether conclusions were supported by results and whether funding and sponsorship may have introduced additional bias. A positive score was given to a study if it was rated yes for the majority of the above questions. Disagreements within the assessments were resolved through consensus.

### 2.6. Data Analysis

Qualitative and quantitative analyses were carried out. For qualitative analysis, we assessed and reported differences in dietary patterns or foods studied and populations characteristics such as ethnicities or geographical areas. For quantitative analysis, statistical measures, including the use of multi-variate controls, dietary intake categorisation, confidence intervals and hazard ratios were assessed along with overall findings of each study. These were then compared with other studies’ findings and grouped in order to analyse the number of consistent or inconsistent findings across studies. Data were considered statistically significant if the reported *p*-value was <0.05. A meta-analysis was not carried out for studies in this review.

## 3. Results

### 3.1. Study Selection 

The literature search process is shown in Figure 1. The initial search resulted in 4698 articles, of which 4461 articles did not fit the inclusion criteria. Seventy-six studies were eligible for full text screening and 46 of these were excluded for the following reasons: abstract only (*n* = 11), included only one nutrient (*n* = 9), wrong article type or study design (*n* = 4), too broad such as studying overall lifestyle habits rather than a focus on diet (*n* = 16), looking at MAFLD not HCC (*n* = 1) and looking at serum/biomarkers (*n* = 5). There were 30 observational studies included in the systematic review [4,7,10,14,20,21,22,23,24,25,26,27,28,29,30,31,32,33,34,35,36,37,38,39,40,41,42,43,44,45].

### 3.2. Study Characteristics 

**Figure 2:**
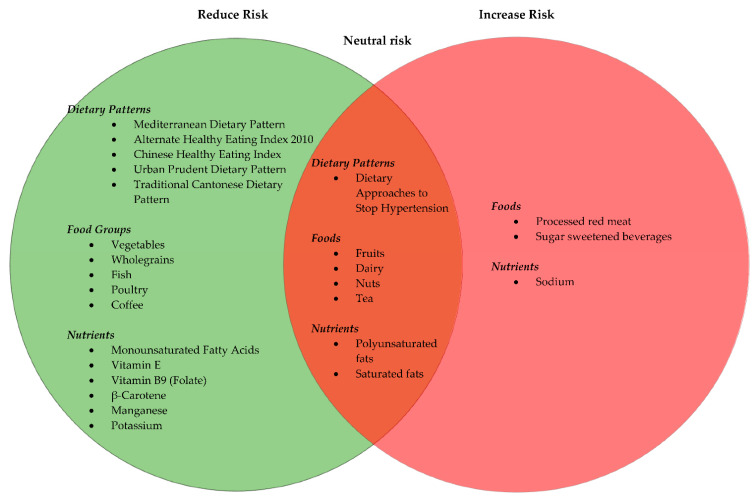
A summary of dietary patterns, food groups and nutrients and the associated risk with hepatocellular carcinoma based on observational studies included in this systematic review.

The data extracted from the included 30 articles are presented in Table 3. All studies were observational and of these: seventeen were cohort, [20,21,22,27,28,29,30,31,32,34,35,38,41,42,43,44,45]; seven were case–control, [4,14,23,33,37,39,40] and six were cohort with nested case–control subset [7,10,24,25,26,36]. In total the 30 studies included 5,222,534 participants aged between 25–85 years across 22 countries including Asian, American and European populations. There were differences according to geographical regions and dietary patterns and components within this review. In particular, the MED pattern indicated protective effects amongst European and American populations [14,30,32]. The Chinese Healthy Eating Index and the Cantonese Dietary Pattern in Asian countries where they are habitually consumed were associated with lower HCC prevalence [23,39]. High-fat dairy products such as butter were associated with HCC prevalence within the American population [25,44] whereas no associations with dairy consumption were observed in the European population [37]. Asian countries such as Japan, who are amongst the highest consumers of soy food [7], indicated lower rates of HCC with increased soy and tofu intake [7]. There appeared to be no differences in food groups including red meat, white meat and fish, vegetables, fruits and coffee intake based on geography. The study periods ranged from 2 to 32 years. The study follow-up period ranged from 4 to 32 years. Four out of 30 studies did not report follow-up periods [23,37,39,40]. Twenty-three out of thirty studies specified HCC as the main type of PLC [4,7,14,20,21,22,24,25,26,27,28,29,30,31,32,33,35,36,37,38,40,43,44] and of remaining seven studies, five reported >85% of participants with HCC [10,23,34,39,42]. The remaining two studies referred generally to PLC [41,45]; however, given the known rates it is likely the majority comprised of HCC cases. The articles were published between 2000 and 2020. Figure 2 summarises the dietary patterns, food groups and nutrients and their associated risk with HCC based on this review. 

### 3.3. Dietary Guidelines and Dietary Patterns

Six studies assessed and identified that there was a relationship between a priori dietary patterns as determined by dietary indices and the risk of HCC [14,22,23,30,32,39]. From this group, two studies were conducted in South China [23,39], one in North-Eastern Italy and Athens, Greece [14], and three in the United States [22,30,32], with a total participant count of 842,270. Adherence to the Mediterranean dietary pattern is reported to be protective against HCC in several studies [14,22,30,32]. A statistically significant association was found between the Mediterranean score and incidence of HCC, with a score of greater than or equal to five (scored out of nine) demonstrating a significant reduction in the risk of HCC when compared with Mediterranean score less than or equal to three (ORs = 0.51, 95% CI: 0.34–0.75, *p* < 0.001) [27] and (HR = 0.62; 95% CI: 0.47–0.84; P trend = 0.0002) [30]. Additionally, the Alternate Mediterranean Diet (aMED) score was associated with a non-significant lower risk of HCC (HR = 0.75; 95% CI, 0.49–1.15; P trend = 0.18) [32]. The aMED score is an adaption of the original Mediterranean diet score, based on the intake of 9 items including vegetables, legumes, fruit and nuts, dairy, cereals, meat and meat products, fish, alcohol and monounsaturated to saturated fatty acid ratio [46]. The aMED score also includes nine components, excluding potato products, separating fruits and nuts into two groups, removes the dairy group, includes whole-grains products, red and processed meats and assigns alcohol intake. The aMED also takes into consideration the chronic disease risk [46,47]. The Chinese Healthy Eating Index and the Healthy Eating Index-2015 (HEI-2015) are designed to assess adherence to the 2016 Dietary Guidelines for the Chinese population and the 2015–2020 Dietary Guidelines for the USA population, respectively, with higher scores (0–100) indicating better adherence to the guidelines. Higher adherence to Chinese or American Dietary Guidelines was significantly associated with lower risk of HCC (*p* < 0.001) [23] and (*p* = 0.03) [30]. The Alternative Healthy Eating Index-2010 (AHEI-2010) is based on the original HEI and includes additional dietary components that predict chronic disease risk, with higher scores (0 to 110 points) associated with lower risk of incident HCC. Greater adherence to the AHEI-2010 was significantly associated with a reduced risk (HR = 0.61, 95% CI: 0.39–0.95, P trend = 0.03) [32]. Similarly, the Urban Prudent Dietary Pattern and the Traditional Cantonese Dietary Pattern were associated with significantly decreased risk of HCC (*p* < 0.002), while the High Meat and Preserved Food Pattern was associated with increased HCC risk (*p* < 0.001). Additionally, a null association was reported between Dietary Approaches to Stop Hypertension Diet and HCC risk (HR = 0.90, 95% CI: 0.59–1.36, *p* = 0.61) [32].

#### 3.3.1. Vegetables and Fruits

High consumption of vegetables with a 100 g/day increment in intake [20] or more than 3–4+ vegetables per week [38] has shown a non-significant trend to reduced risk of HCC [20,38,45]. However, no associations were seen with fruit consumption [20,37,43,45]. A specific subgroup of vegetables including celery (*p* = 0.03), mushrooms (*p* = 0.03), allium vegetables (Chinese chives, onions, garlic, garlic shoots) (*p* < 0.01), composite vegetables (asparagus-lettuce, garland chrysanthemum) (*p* < 0.01), legumes and legume products (*p* = 0.04), squash and carrots, had a significant inverse association, indicating protective effects against HCC [45]. Potato intake was associated with reduced HCC mortality in women, whereas frequent intake of potatoes cooked in soy sauce increased HCC mortality in men [38]. 

#### 3.3.2. Red Meat, White Meat and Fish 

Processed meat and red meat intake were associated with an increased risk of HCC [4,31]. A positive association was found between processed red meat and HCC risk, where higher contribution to total calorie intake from processed red meat (comparing highest to lowest tertile intake) indicated significant findings, reporting an 84% increased HCC risk (HR = 1.84, 95% CI: 1.16–2.92, *p* = 0.04) [31]. 

Conversely, higher intake (3.5 servings/week) of white meat demonstrated a 39% lower risk of HCC (comparing highest to lowest tertile intake, HR = 0.61, 95% CI: 0.40–0.91, *p* = 0.02) [31] and a protective association (HR = 0.52, 95% CI: 0.36–0.77) with HCC incidence [27]. USA cohort studies, the Nurses’ Health Study (NHS) and Health Professionals Follow-up Study (HPFS) further examined the type of white meat (i.e., poultry) intake, reporting a significantly protective association (HR = 0.60, 95% CI: 0.40–0.90, *p* = 0.01) with HCC [31]. However, some studies did not report any association [26,31]. Greater consumption of fish was associated with reduced risk of HCC [26,31]. Each daily 20 g of fish consumption correlated with a reduction in HCC development (HR = 0.80, 95% CI: 0.69–0.97). [26] The European Prospective Investigation into Cancer and Nutrition (EPIC) and, NHS and HPFS studies reported that substituting 20 g/day in place of fish for meat resulted in a 16% decrease in HCC risk [26], and substitution of poultry or fish for processed red meat was associated with a decrease in risk of HCC (HR = 0.79, 95% CI:0.61–1.02) [31]. 

#### 3.3.3. Dairy and Soy

Two large cohort studies, one conducted in the USA and the other using data from EPIC and NHS, and HPFS showed that higher total dairy product intake was associated with a statistically significant higher risk of HCC [25,44]. However, the association differed by the type of dairy products consumed [30]. Higher intake of dairy products (>381.7 g/day) showed increased HCC risk in the EPIC study (highest vs. lowest tertile, HR = 1.66, 95% CI: 1.13–2.43, P trend = 0.012) [25], and NHS and HPFS study (HR = 1.85, 95% CI: 1.19–2.88; *p* = 0.009) [44]. Additionally, significant positive HCC risk association was observed for high-fat dairy (*p* = 0.008), butter (*p* = 0.04) and milk (P trend = 0.049) [25,44]. Intake of yoghurt showed a trend to lower HCC risk [44] and indicated no association in another study [25]. This discrepancy may be attributed to the differences in the content of insulin-like growth factor (IGF-1) and aflatoxin in milk, cheese and yoghurt. Although the European Food Safety Authority reports low aflatoxin M_1_ levels in milk samples; due to the high consumption of milk in Europe, the daily ingestion of aflatoxin M_1_ remains significant [25]. Conversely, no significant associations were found between dairy consumption and HCC risk based on the results of two case–control studies [4,37]. Increased intake of soy foods was found to reduce risk of HCC in a cohort-based, nested case–control study conducted within the Japanese population [7]. Intake of miso soup (>17.1 g/day) or tofu (>76.3 g/day) more than 5 times/week was associated with 50% lower HCC risk, when compared to less than once a week [7]. This reduction in crude HCC risk was 0.89 for miso soup and 0.92 for tofu, per additional serving [7]. Increased intake of dairy products, particularly high fat, appears to be associated with increased risk of HCC. However, there is much heterogeneity in types and quantity of consumption thus, further studies are warranted.

#### 3.3.4. Wholegrains 

Wholegrain intake is associated with decreased HCC risk (highest versus lowest tertile: HR = 0.63, 95% CI: 0.41–0.96, *p* = 0.04) with daily intake range of 17.86–33.28 g/day [4,43]. Surprisingly, a significant positive association was found between cereal intake and HCC risk (highest versus lowest tertile OR = 1.87, 95% CI: 1.09–3.22, *p* = 0.035) in the CiRCE study [4]. The dietary glycemic index, glycemic load, and carbohydrate intake did not find any association with HCC in a Chinese population [42]. 

#### 3.3.5. Nuts

In two large USA prospective cohorts, NHS and HPFS, higher total nut (HR = 0.84, 95% CI, 0.56–1.26), walnuts (*p* = 0.19) and peanuts (*p* = 0.90) consumption was not strongly associated with HCC risk [35]. Whereas, an increased intake (mean 1.25 serving per week) of tree nuts (including hazelnuts, almonds, macadamias, pecans, cashews and pistachios) reported a suggestive association with lower HCC risk (HR = 0.64, 95% CI: 0.43–0.95) [35]. Overall, nut consumption did not indicate a strong association with HCC risk.

#### 3.3.6. Beverages 

##### Coffee

Coffee consumption has been shown by a number of studies to be associated with reduced risk of HCC incidence [21,28,29,34,38,41]. The Takayama Study revealed that coffee intake twice per day or more had a significantly lower risk of HCC when compared with non-drinkers (HR = 0.4, 95% CI: 0.20–0.79, *p* = 0.03) [41]. Daily coffee drinkers had a 51% lower HCC risk than those who abstained (HR = 0.49, 95% CI: 0.36–0.66, *p* < 0.001) [28], and a dose response was shown with those consuming greater than two cups/day having a statistically significant reduction in risk of HCC (*p* = 0.49) [34]. Consumption of decaffeinated coffee showed no significant association with HCC risk [41].

##### Tea

The MEC study demonstrated that increased tea intake (>475.1 mL/day for females, >480 mL/day for males) was associated with lower HCC risk (HR = 0.41, 95% CI: 0.22–0.78, *p* = 0.003) [21]. Other studies investigated in this review did not report any association between tea and the risk of HCC [29,38,40,41]. 

##### Sugar-Sweetened Beverages 

A positive association between HCC risk and carbonated/soft drink beverages was seen in large Asian and European cohorts (OR = 2.44, 95% CI: 1.17–5.09, *p* = 0.021) [4]. Compared to non-consumers, consumption of >6 servings/week (6 × 330 mL can) of soft drinks was significantly associated with higher HCC risk (HR = 1.83, 95%CI: 1.11–3.02, *p* = 0.01) [10]. Consumption of juice less than 200 mL glass a week was associated with lower HCC risk (HR = 0.60, 95% CI: 0.38–0.95, *p* = 0.02) when compared to non-consumers [10]. 

##### Alcohol 

Alcohol was reported as a major risk factor for HCC. However, many studies failed to show a significant positive association with HCC risk [4,7,10,14,20,21,22,23,24,25,26,27,28,29,33,34,36,37,38,39,40,41,42,43,45]. This was because the relationship between alcohol consumption and HCC is difficult to interpret, as the risk often depends on the quantity and duration of alcohol consumption along with other factors such as age, gender, presence of viral hepatitis, cirrhosis and metabolic syndrome [4]. In the methodology of most studies, alcohol was reported as a potential confounding factor and was adjusted. 

#### 3.3.7. Macronutrients

##### Fats

Monounsaturated fats are associated with reduced HCC risk, as shown in a large prospective European cohort (HR = 0.71, 95% CI: 0.55–0.92) with intake range from 22.05 to 43.35 g/day, and a similar study in Greece (OR = 0.47, 95% CI: 0.25–0.87) [24,37]. Saturated fat in red meat increased the risk of HCC (HR = 1.87, 95% CI: 1.23–2.85); suggesting that the association with red meat may be as a result of saturated fat [27]. On the contrary, no direct association of HCC risk with saturated fat intake was shown in case-control study from Italy [36]. A strong protective association was identified between HCC risk and polyunsaturated fatty acids (OR = 0.48, 95% CI: 0.24–0.94) with the effect postulated to be due to linoleic acid (OR = 0.35, 95% CI: 0.18–0.69, *p* < 0.01) [33]. Omega-6 PUFA intake demonstrated a significant dose-dependent, positive association with HCC risk in Singaporean Chinese (HR = 1.49, 95% CI: 1.08–2.07, *p* = 0.02) and omega-3 PUFA conferred no association [36]. Overall, the data suggest that monounsaturated and polyunsaturated fatty acids were associated with reduced HCC risk in comparison to saturated fats, which displayed no impact or positive HCC risk associations. 

#### 3.3.8. Micronutrients 

Vitamin E (*p* = 0.017), vitamin B9 (folate) (*p* = 0.036), β-carotene (*p* = 0.03), manganese (*p* = 0.038) and potassium (*p* = 0.004) in the diet have shown a significant negative correlation with HCC risk [4,38]. Sodium intake was significantly associated with an increased HCC risk (*p* = 0.043) [4]. Dietary iron (mean: 13.9 mg/day) intake was associated with increased HCC risk (*p* = 0.01); however, the association was weakened when contribution of wine was excluded [33]. Thus, it may be difficult to come to a conclusion due to confounding factors such as wine and red meat intake. 

### 3.4. Risk of Bias 

The risk of bias of the included observational studies was assessed by The Academy of Nutrition and Dietetics Evidence Analysis Library (EAL) Quality Criteria Checklist and is outlined in Table 4. The Quality Criteria Checklist: Primary Research has ten validity questions based on the Agency for Healthcare Research and Quality domains for research studies. Studies can score a positive (+) which indicates that the report has addressed issues of inclusion/exclusion, bias, generalisability, and data collection and analysis, negative (−) which indicates issues were not addressed adequately, or neutral [48] which indicates that the study is neither strong nor weak. Overall, the included studies were considered to be at low risk of bias as they showed a positive quality rating.

## 4. Discussion

To our knowledge, this is the first robust and most up-to-date systematic review following the PRISMA guidelines, evaluating the association between diet and dietary patterns and HCC risk. The results show that diet plays an important role in HCC occurrence. Consumption of dietary patterns such as the MED pattern, the AHEI-2010, the Urban Prudent Dietary Pattern and the Traditional Cantonese Dietary Pattern, foods such as vegetables, poultry, fish, wholegrains, and coffee, and micronutrients such as vitamin E, vitamin B9, β-carotene, manganese and potassium may have a potential benefit in reducing the development of HCC. Some fats, including monounsaturated fats, may also have beneficial effects. Additionally, it can be inferred that sugar-sweetened beverages including soft drinks/carbonated beverages and processed red meat consumption may increase HCC risk. Whilst some dietary patterns including the DASH diet and foods such as nuts (e.g., walnuts and peanuts) indicate a null association. 

Some of the important risk factors for HCC have been modified over the last decade, including the treatment of chronic hepatitis B and hepatitis C viruses with direct acting antiviral agents, and it is expected that the rate of HCC related to these factors will decrease. However, increasingly HCC is related to obesity and fatty liver, and with the current obesity epidemic the incidence is not likely to improve. It is possible that the changes in diet and dietary patterns that are related to the increase in obesity may also be having a direct effect on HCC pathogenesis. Some of these mechanisms were included in studies such as the reduction in HCC risk with intake of yellow vegetables in patients with hepatitis B (HBV) or hepatitis C (HCV) infection [49]. The carotenoids, in particular β-carotene, have been shown to neutralise free radicals in the liver tissue, thus decreasing carcinogenesis in patients with hepatitis and preventing the progression of HBV and HCV related HCC [49,50]. 

Fruit and vegetable intake are associated with a lower incidence of conditions such as obesity, diabetes and other cancers [20,33,51,52]. Surprisingly, our review found no consistent relationship between fruit intake and HCC. This may be as a result of the observational study designs and different methods used to obtain dietary information. 

Meat intake is affected by important confounders, such as gender, body mass, smoking and HBV and HCV status [53]. Red meat has high amounts of carcinogens, including haem iron, which in excess induces fibrosis and cirrhosis [33,54]. Red meat also has high cholesterol and saturated fat content, related to known cancer risk factors such as obesity, diabetes and metabolic syndrome. In addition to this, processing and preservation of meat forms potential carcinogenic chemicals such as N-nitroso compounds [53]. By contrast, fish and poultry are lower in saturated fat and cholesterol and are higher in polyunsaturated fatty acids, which inhibit tumour-necrosis factor and inflammation; playing a protective role in hepatocarcinogenesis [53,55]. 

Omega-6 polyunsaturated fatty acids may increase HCC risk by their association with individuals who are overweight, obese and/ or have diabetes [36]. Additionally, the metabolism of omega-6 polyunsaturated fatty acid produces pro-inflammatory products including lipid mediators and indirectly C-reactive protein; which have been implicated in causing fibrosis in MAFLD, subsequently cirrhosis and ultimately HCC [24,27,55]. Another consideration is that the sources of monounsaturated fats in the United States are generally from meat and/or meat products compared to Europe where the main sources are oils and added fats [56,57]. 

Dairy consumption has been associated with several cancers such as gastric cancer [19]. This may at least be part attributable to the presence of saturated fat, IGF-1 and other contaminants [58]. Potential carcinogenic contaminants include bracken fern, which is added to feedstuff and passed into milk [58]. Calcium and vitamin D found in dairy products may also increase the circulation of IGF-1, which plays an important role in cell proliferation and carcinogenesis [25], leading to the development of breast cancer [59]. Future studies are recommended in experimental and prospective settings. 

In the Japanese population, weekly consumption of phytoestrogen, isoflavone, present in large amounts in miso and tofu revealed a 50% reduction in HCC risk [7]. Isoflavone is believed to interact with estrogen, inhibiting its effect on cell proliferation and inducing apoptosis [7]. Miso and tofu consumption in a Japanese population was quantified, but did not include tofu included in mixed meals or natto, another soy product consumed in the common Japanese diet, resulting in possible underestimation of the effects of tofu [7]. Given the large popularity of fermented soy products in Japanese culture, the difference observed between the quartiles in this study were small, warranting further research in non-Japanese populations. 

The varying results on wholegrain consumption can be attributed to the different sample sizes, demographics, and cultural differences between American and European populations [4,43]. Moreover, the different study designs (i.e., case control versus cohort) and the dietary assessment methods may also be a possible explanation for the unexpected results. Nuts are nutrient-dense foods rich in unsaturated fats, vegetable protein, vitamins, folate, fiber, and minerals [35]. Although studies do not support a strong association with higher nut (e.g., walnuts and peanuts) consumption and reduced HCC risk, it is plausible that higher intake might influence HCC risk through mechanisms related to insulin resistance and inflammation [35,60]. Nuts are associated with lower risk of type II diabetes, a risk factor for HCC [61].

High carbohydrate intake leads to a high circulation of endogenous insulin, and thus insulin influencing IGF-1, a known carcinogen [62]. However, it remains unclear whether carbohydrates affect the risk of developing HCC specifically. Sugar-sweetened beverages, juices, and soft drinks consumption are associated with overall cancers including HCC [63]. This may be explained by the effect of sugary drinks on weight gain and obesity [63], but also hyperinsulinaemia and type 2 diabetes, thus increasing the risk of diabetes-related carcinomas (liver, pancreas, breast) [63]. The high glucose and fructose cause a rapid increase in insulin levels and accelerate the formation of fat in the liver [64]. It is also likely that people who consume high levels of sugar-sweetened beverages have poorer diet quality [10].

Coffee constituents such as chlorogenic acid may account for the hepato-protective effects of coffee with HCC [48]. It also contains antioxidants and phenolic compounds which have anticarcinogenic properties [65].

The Mediterranean diet has been shown to have benefits in health, longevity and decreased mortality. The diet exerts anti-inflammatory effects as it is lower in saturated fat, refined sugar and dairy, and higher in unsaturated fatty acids, fruits and vegetables, wholegrains and fish [65,66,67]. Foods consumed in the Chinese culture are associated with a lower HCC risk which may be due to the higher consumption of soy products, seafood, traditional soups and herbal teas, possibly via the increased antioxidants in these foods [23,39]. 

This systematic review had several notable strengths, with an overall positive risk of bias assessment score (Table 4). Data were synthesized from twenty-one different countries across Europe, Asia and North America, enrolling a large sample size of participants. Additionally, twenty-six out of 30 studies used a validated tool to collect and assess dietary information, including food frequency questionnaires, diet history questionnaires, country-specific questionnaires and centre-specific questionnaires. The limitations arise from using observational studies, predominantly cohort and case–control design; and that the food frequency questionnaires were conducted at a single point in time to assess long-term usual diet. Another limitation stems from the lack of evidence available from prospective studies, where more high-quality studies including generalisable populations assessing the association between HCC risk and diet and dietary pattern over time are required to affirm the findings from this review. Additionally, dietary studies are complicated by virtue and it remains challenging for researchers to interpret diet holistically. For example, consumption of one food is perceived to lead to adverse health outcomes, such as processed meat; however, it is hard to understand whether this was a result of an overall poor diet or the role of processed meat itself. The complex interconnections between foods, nutrients and dietary patterns imply that no individual element can provide a complete picture on nutrition and thus health status. The whole diet approach i.e., dietary patterns, which incorporates a combination of food groups, have been demonstrated to be beneficial and in fact more robust compared to assessment of individual nutrients. The reason for this being, that dietary patterns represent how foods are consumed, which is not in isolation as individual nutrients, rather as whole foods in meals and as such dietary patterns better account for the synergistic effect of the food matrix [68,69,70]. However, there were only a few studies included within this systematic review that assessed dietary patterns.

## 5. Conclusions

Current epidemiological evidence supports that diet and dietary patterns are relevant factors related to the risk of HCC. Certain dietary patterns including the Mediterranean diet, the Alternative Healthy Eating Index-2010, the Urban Prudent Dietary Pattern, the Traditional Cantonese Dietary Pattern, foods including vegetables, wholegrains, fish, poultry, coffee, macronutrients including monounsaturated fatty acids, and micronutrients including vitamin E, vitamin B9, β-carotene, manganese and potassium may lead to reduced risk of HCC. This provides a basis for determining what types of dietary interventions may reduce the development of HCC. However, what the benefits are to high-risk individuals such as those who already have HBV or cirrhosis remains unknown. Future prospective studies should be well-designed and include large and diverse geographical regions including participants with differing socioeconomic status-, ethnic-, race-based populations. Heterogeneity between high-risk populations (e.g., high-risk family cohorts, patients with cirrhosis) should be included and characterized, as should family history, genetics, and other modifiable lifestyle factors (e.g., physical activity) to ensure data account for high-risk groups and are more widely representative and generalisable. Future studies may also consider examining diets holistically through assessment into dietary patterns. Healthier dietary patterns may reduce the prevalence of HCC risk based on the findings from this study, albeit the current literature is limited. Additionally, studies should consider investigating key dietary patterns (including traditional dietary patterns) such as the MED, the AHEI-2010, the Cantonese Dietary Pattern and DASH as shown by Lan et al. 2018 [39] and Ma et al. 2019 [32]. It appears, based on our assessment of dietary patterns into geographical regions, that selecting a pattern that best reflects the traditional, habitual dietary intake of a given population is most appropriate. Researchers may consider expanding to other dietary patterns as applicable, which are shown to be protective for disease prevention, but not yet HCC such as vegetarian/vegan, plant-based diets and Nordic dietary patterns may also be worth exploring to elucidate HCC risk. As components of all lifestyle behaviours are integrated, designing studies that correlate all aspects of a healthy diet are recommended.

## Figures and Tables

**Table 1:** PICOS criteria for inclusion and exclusion of studies.

Parameter	Criteria
Population	Adults of both sexes above the age of 18 years
Intervention	Different dietary patterns (e.g., Mediterranean, Prudent), key food groups and nutrients
Comparison	Those who do not develop hepatocellular carcinoma are compared to individuals who do
Outcome	Risk of hepatocellular carcinoma
Study Design	Observational studies (including case–control studies, nested case–control studies or cohort studies)

**Table 2:** Risk of bias questions.

Relevance questions
1. Would implementing the studied intervention or procedure (if found successful) result in improved outcomes for the patients/clients/population group?
2. Did the authors study an outcome (dependent variable) or topic that the patients/clients/population group would care about?
3. Is the focus of the intervention or procedure (independent variable) or topic of study a common issue of concern to dietetics practice?
4. Is the intervention or procedure feasible?
**Validity questions**
1. Was the research question clearly stated?
2. Was the selection of study subjects/patients free from bias?
3. Were study groups comparable?
4. Was method of handling withdrawals described?
5. Was blinding used to prevent introduction of bias?
6. Were intervention/therapeutic regimens/exposure factor or procedure and any comparison (s) described in detail? Were intervening factors described?
7. Were outcomes clearly defined and the measurements valid and reliable?
8. Was the statistical analysis appropriate for the study design and type of outcome indicators?
9. Are conclusions supported by results with biases and limitations taken into consideration?
10. Is bias due to study/s funding or sponsorship unlikely?

**Table 3:** Summary of studies (*n* = 30) evaluating the association between diet and hepatocellular carcinoma risk.

Author; Study Period	Study Design	Country(ies) of Study	Sample Size; Male and Female; Disease State	Participant Characteristics: Age (years); BMI (kg/m^2^); Co-Morbidities	Diet	Dietary Assessment Method	Risk of HCC
EPIC Cohort studies							
Stepien et al. (2016) [10] 1992–1998	European Prospective Investigation into Cancer and Nutrition (EPIC) Cohort study	Denmark, France, Greece, Germany, Italy, the Netherlands, Norway, Spain, Sweden, and the United Kingdom	*n* = 477,206 M: 141,945 F: 334,768 HCC cases: 191	Mean age: 59.6 Mean BMI: 28.0 Diabetes: 11.5%	Soft drinks, fruit and vegetable juices	Country-specific dietary questionnaires	Soft drink (>6 servings/week): HR = 1.83, 95% CI: 1.11–3.02, *p* = 0.01 Artificially sweetened soft drinks: HR = 1.06, 95% CI: 1.03–1.09 Sugar-sweetened soft drinks: HR = 1.00, 95% CI: 0.95–1.06 Juice (<1 serving/week): HR = 0.60, 95% CI: 0.38–0.95, P trend = 0.02
Bamia et al. (2015) [20] 1992–2010			*n* = 486,799 M: 145,039 F: 341,760 HCC cases: 201	Mean age: 49.7 Diabetes (self-reported): 14.8%	Fruit and vegetable intake	Centre-specific questionnaires	Higher vegetable intake: HR = 0.83, 95% CI: 0.71–0.98Fruit intake: HR = 1.01; 95% CI: 0.92–1.11
Bamia et al. (2015) [21] 1992–2010			*n* = 486,799 M: 145,039 F: 341,760 HCC cases: 201	Mean age: 53 Diabetes (self-reported): 47.5%	Coffee, tea and decaffeinated coffee intake. Median coffee consumption: M: 354 mL/d F: 290 mL/d		High coffee consumers (Q5 vs. Q1): HR = 0.28, 95% CI: 0.16–0.50, *p* < 0.001 Decaffeinated coffee: HR = 0.94, 95% CI, 0.39 to 2.28 High tea consumers vs. low tea consumers: HR = 0.41, 95% CI: 0.22–0.78, *p* = 0.003
Duarte- Salles et al. (2015) [24] 1992–2010			*n* = 477,206 M: 142,194 F: 335,012 HCC cases: 191	Mean age: 50.6 Mean BMI: 25.3 Diabetes: 2.3%	Total dietary fat, Subtypes of fats (monounsaturated, polyunsaturated and saturated) and sources of fats (added fats, meat and meat products and dairy products)	Country-specific dietary questionnaires	Total fat (highest vs. lowest): HR = 0.80, 95% CI: 0.65–0.99 Monounsaturated fat: HR = 0.71, 95% CI: 0.55–0.92 Saturated fats: HR = 1.08, 95% CI: 0.88–1.34
Duarte-Salles et al. (2014) [25] 1992–2010			*n* = 477,206 M: 142,194 F: 335,012 HCC cases: 191		Milk, cheese and yogurt		Total dairy products (highest vs. lowest tertile): HR = 1.66, 95% CI: 1.13–2.43, *p* = 0.012 Milk (highest vs. lowest tertile): HR = 1.51, 95% CI: 1.02–2.24, *p* = 0.049 Cheese (highest vs. lowest tertile): HR = 1.56, 95% CI: 1.02–2.38, *p* = 0.101 Yogurt (highest vs. lowest tertile): HR = 0.94, 95% CI: 0.65–1.35, *p* = 0.848
Fedirko et al. (2013) [26] 1992–2010			*n* = 477,206 M: 142,194 F: 335012 HCC cases: 191		Total meat and fish		Total fish intake: HR = 0.80, 95% CI: 0.69–0.97 Lean fish (per 10 g/day): HR = 0.91, 95% CI: 0.81–1.02) Fatty fish (per 10 g/day): HR = 0.92, 95% CI: 0.82–1.03) Crustaceans and molluscs: HR = 0.86, 95% CI: 0.70–1.06 20 g increase in total meat intake offset by a decrease in total fish intake: HR = 1.16, 95% CI: 1.01–1.34 Total meat: HR = 0.93, 95% CI: 0.82–1.12 per 20 g/day Red/processed meats: HR = 0.95, 95% CI: 0.88–1.06 per 10 g day Poultry: HR = 0.99, 95% CI: 0.91–1.09 per 10 g/day
MEC Cohort studies							
Bogumil et al. (2019) [22] 1993–2013	Multi-ethnic centre (MEC) cohort study	United States California and Hawaii	*n* = 169,806 HCC cases: 605	Age range: 45–75 Mean BMI: 27.2 Diabetes: 8.4%	Healthy Eating Index- 2010 Alternative Healthy Eating Index-2010Alternate Mediterranean Diet Dietary Approaches to Stop Hypertension	FFQ	Healthy Eating Index-2010 (Q5 vs. Q1): HR = 0.69, 95% CI: 0.53–0.91; *p* = 0.003 Alternative Healthy Eating Index-2010 (Q5 vs. Q1): HR = 0.74, 95% CI: 0.58–0.95; *p* = 0.048 Alternate Mediterranean Diet (Q5 vs. Q1): HR = 0.68, 95% CI: 0.51–0.90; P trend = 0.016 Dietary Approaches to Stop Hypertension (Q5 vs. Q1): HR = 0.80, 95% CI: 0.62–1.03; *p* = 0.045
Setiawan et al. (2015) [34] 1993/1996–2012/2012			*n* = 162 022 HCC cases: 451		Coffee intake		1 cup coffee/day: RR = 0.87, 95% CI: 0.67–1.11 2–3 cups coffee/day: RR = 0.62, 95% CI: 0.46–0.84 ≥4 cups of coffee/day: RR = 0.59; 95% CI: 0.35–0.99
Sun-Yet Sun University Centre case–control studies							
Chen et al. (2018) [23] Sep 2013–Oct 2017	Sun-Yet Sun University Centre case–control study	China	HCC cases: 720 M: 613 F: 107 Control: 720 M: 613 F: 107	Mean age: Cases: 58.2 Controls: 58.4 Mean BMI: Control: 23.7 Cases: 22.8 Diabetes: Cases: 76 Controls: 57	The Chinese Healthy Eating IndexThe Healthy Eating Index-2015	FFQ	The Chinese Healthy Eating Index OR = 0.43, 95% CI: 0.38–0.50 Healthy Eating Index-2015: OR = 0.47, 95% CI: 0.40–0.55
Lan et al. (2018) [39] Sep 2013–Aug 2016			HCC cases: 782 M: 680 F: 102 Control: 782 M: 680 F: 102	Mean age: 58 Cases: 52.7 Control: 53.02 Mean BMI: Cases: 22.81 Control: 23.25 HTN: Cases: 14.2% Controls: 11.8% Diabetes: Cases: 8.6% Controls: 4.9%	Urban Prudent Dietary Pattern Meat and Preservative Dietary Pattern Traditional Cantonese Dietary Pattern		Urban Prudent Dietary Pattern (highest quartile): OR = 0.25, 95% CI: 0.18–0.35, *p* = <0.001 Meat and Preservative Dietary Pattern (highest quartile): OR = 1.98, 95% CI: 1.46–2.6, *p* <0.001Traditional Cantonese Dietary Pattern (highest quartile): OR = 0.61, 95% CI: 0.46–0.82; P trend = 0.002
Singapore Chinese Health Study Cohort studies							
Koh et al. (2016) [36]	Singapore Chinese Health Study cohort study	Southern China	*n* = 60,298 HCC: 488 F: 134 M: 354	Age range: 45–74 Mean BMI: Cases: 23.9 Non-cases: 23.1 Diabetes: Cases: 18.0% Non-cases: 8.8%	Fatty acids (saturated, monounsaturated, omega-3 and omega-6 PUFA)		Omega-6 PUFA intake (top quartile): HR = 1.49, 95% CI: 1.08–2.07
Johnson et al. (2011) [29] 1993–1998		China	*n* = 61,321 HCC cases: 362		Coffee, black tea, and other types of tea (e.g., green tea)		Coffee (3+ cups per day vs. non-drinkers): HR = 0.56, 95%C I: 0.31–1.00, *p* = 0.049
Aviano National Cancer Institute case–control studies							
Montella et al. (2007) [40] Jan 1999–Jul 2002	National Cancer Institute in Aviano, the ‘Santa Maria degli Angeli’ General Hospital in Pordenone, the ‘Pascale’ National Cancer Institute, and four General Hospitals in Naples Case–control study	Province of Pordenone (north- eastern Italy) and city of Naples (southern Italy)	HCC cases: 185 M: 149 F: 36 Controls: 412 M: 281 F: 131	Age range: 43–84	Coffee, decaffeinated coffee and tea		Coffee consumption (≥28 cups/week): OR = 0.43, 95%CI: 0.16–1.13, *p* = 0.02 Decaffeinated coffee: OR = 0.72, 95%CI: 0.21–2.50 Tea (≥1 cup/week): OR = 1.43, 95% CI: 0.76–2.66
Polesel et al. (2007) [33] Jan 1999–Jul 2002			HCC cases: 185 M: 149 F: 36 Controls: 412 M: 281 F: 131	Age range: 43–84	Dietary data divided into 7 sections: milk, hot beverages and sweeteners; bread, cereals; first courses; second courses (meat and other mains); side dishes (vegetables); fruits; sweets, desserts and soft drinks; alcoholic beverages		High iron-containing foods: OR = 3.00, 95% CI: 1.25–7.23 Wine:OR = 1.61, 95% CI: 0.78–3.30 Polyunsaturated fatty acids: OR = 0.35, 95% CI: 0.18–0.69 B-carotene also possibly reduces HCC risk (OR = 0.48, 95% CI: 0.24–0.93).
Shanghai Women’s Health Study and Shanghai Men’s Health cohort study							
Vogtman etal. (2013) [42]	Shanghai Women’s Health Study and Shanghai Men’s Health Study cohort study	Shanghai, China	*n* = 132,837 M: 60,207 F: 72,966	Mean age: M: 54.8 F: 50.7 Mean BMI: M: 23.7 F: 23.7	Dietary glycemic index, glycemic load and carbohydrate		Consumption of glycemic load: Women Q5: HR = 1.13, 95% CI: 0.66–1.93 Men Q5: HR = 1.07, 95% CI: 0.70–1.66 Consumption of glycemic Index: Women Q5: HR = 2.41, 95% CI: 1.23–4.7 Men Q5: HR = 0.95, 95% CI: 0.63–1.43 Consumption of carbohydrate: Women Q5: HR = 0.92, 95% CI: 0.56–1.50 Men Q5: HR = 1.16, 95% CI: 0.75–1.81
Zhang et al. (2013) [45]			*n* = 132,837 M: 60,207 F: 72,966 HCC: 267	Mean age: M: 56.1 ± 10.3 F: 52.6 ± 9.1 Mean BMI non-cases: M: 23.7 ± 3.1 F: 24.0 Mean BMI cases: M: 23.3 ± 0.3 F: 24.7 ± 0.3	Vegetable-based diet, fruit-based diet and meat-based diet		Vegetable-based dietary pattern (Q4): HR = 0.58, 95% CI: 0.40–0.84; *p* = 0.01 Fruit-based dietary pattern (Q4): HR = 1.13, 95% CI: 0.78–1.64; *p* = 0.39 Meat-based dietary pattern (Q4): HR = 1.18, 95% CI: 0.83–1.69; *p* = 0.51
Freedman etal. (2010) [27] 1995–1997	Cohort study	California, Florida, Georgia, Lousiana, Michigan, New Jersey, North Carolina, Pennsylvania	*n* = 303,172 M: 176,845; F: 126,327 HCC: 338	Age range: 50–71 Mean age: Women—Cases: 59.0 Controls: 52.4 Men—Cases: 59.4 Controls: 55.2 Mean BMI: Women—Cases: 24.7 Controls: 24.0 Men—Cases: 23.3 Controls: 23.7	Red meat, white meat, processed meat and total fat		White meat:HR = 0.52, 95% CI: 0.36–0.77 Red meat: HR = 1.74, 95% CI: 1.16–2.61 Saturated fat: HR = 1.87, 95% CI: 1.23–2.85 Total fat intake (Q5 vs. Q1: HR = 1.46, 95% CI: 0.98–2.19, *p* = 0.045
Inoue et al. (2005) [28] 1990–1993	Cohort study	Japan	*n* = 90,452 M: 43,109 F: 47,343 HCC: 334	Age range: 40–69	Coffee consumption	Self-administered questionnaire	Coffee: HR = 0.49, 95% CI: 0.36–0.66 1–2 cups/day: HR = 0.52, 95% CI: 0.38–0.73 3–4 cups/day: HR = 0.48, 95% CI: 0.28–0.83 ≥5 cups/day: HR = 0.24, 95% CI: 0.08–0.77
Kuper et al. (2000) [37] Jan 1995–Dec 1998	Case–control study	Greece	*n* = 225 Controls: 128 M: 110 F: 18 HCC incidence: 97 M: 85 F: 12	NA	Food groups: cereals; starchy roots; sugars and syrups; pulses and nuts; vegetables; fruits; meats, fish, and eggs; milk and dairy products; added lipids; and non-alcoholic beverages	FFQ	Vegetable intake: OR = 1.21, 95% CI: 0.80–1.82, *p* = 0.36 Dairy intake: OR = 0.70, 95% CI: 0.49–1.01, *p* = 0.06 Monounsaturated fat: OR = 0.47, 95% CI: 0.25–0.87
Kurozawa et al. (2004) [38] 1988–1999	Cohort study	Japan	*n* = 110,688 M: 46,399 F: 64,289	Age range: M: 40–59 F: 40–59 BMI: NA	33 food items: beef, pork, ham and sausage, chicken, liver, eggs, milk, yogurt, cheese, butter, margarine, fried food, fried vegetables, fish, fish paste, dried fish, green leafy vegetables, carrots and squash, tomatoes, cabbage and lettuce, Chinese cabbage, edible wild plants, mushrooms, potatoes, seaweeds, pickles, foods boiled down in soy sauce (tsukudani), boiled beans, tofu, oranges, fruits other thanoranges, fruit juice and cakes	Self-administered questionnaire	Carrots and squash (3–4x/week) women aged 60–79 years: HR = 0.29, 95% CI: 0.10–0.78, *p* < 0.05 Potatoes (1–2x/week) women aged 40–59 years: HR = 0.10, 95% CI: 0.01–0.99, *p* < 0.05 Coffee (1+/day) men aged 60–79 years: HR = 0.41, 95% CI: 0.19–0.90 Coffee (1+/day) women aged 60–79 years: HR = 0.30, 95% CI: 0.10–0.89
Rizk et al. (2019) [4] Jun 2008–Dec 2012	Case– control study	North- East France	*n* = 582 Controls: 401 M: 267 Cases: 181 M: 156	Mean age: Controls: 59 Cases: 64	Food variables were measured for 27 predefined food groups Information about the consumption of 208 food items and 23 nutrients were collected	Diet history questionnaire	Carbonated beverages: OR = 2.44, 95% CI: 1.17–5.09; *p* = 0.021 Total cereals group: OR = 1.87, 95% CI: 1.09–3.22; *p* = 0.035 Processed meat group: OR = 1.97, 95% CI: 1.14–3.41; *p* = 0.028 High-fat dairy products: OR = 1.41, 95% CI: 0.82–2.43; *p* = 0.36 Low-fat dairy products: OR = 1.01, 95% CI: 0.58–1.76; *p* = 0.82
Sharp et al. (2005) [7] 1965–1988	Cohort, with nested case-control subset	Japan	HCC cases: 176Control: 560	NA	Soya food consumption	FFQ	Miso soup (5 times/week): OR = 0.5, 95% CI: 0.29–0.95 Tofu (5 times/week): OR = 0.5, 95% CI: 0.20–0.99 Miso soup (95% CI: 0.80–0.98) Tofu 0.92 (95% CI: 0.81–1.05)
Tamura et al. (2018) [41] Sep 1992–Mar 2008	Cohort study	City of Takayama, Gifu Prefecture, Japan	*n* = 30,824 M: 14,240 F: 16,584	Mean age: 55.3 BMI: 18.5 to <25: 22, 182 History of diabetes: 4.5%	Coffee, green tea, black tea, caffeine and decaffeinated coffee consumption		Coffee (2x/day or more): HR = 0.4, 95% CI: 0.20–0.79, *p* = 0.03
Turati et al. (2014) [14] 1999–2002 and 1995–1998	Case–control study	Province of Pordenone, city of Naples and Athens, Greece	HCC cases: 518 M: 432 F:86 Control: 722 M: 579 F: 193	Mean age: Cases: 66 Controls: 65	Mediterranean diet		Mediterranean Diet Score: ORs = 0.51, 95% CI: 0.34–0.75, *p* < 0.001
Yang et al. (2019) [43] 1984–2012 and 1986–2012	Cohort study	United States	*n* = 125,455 M: 48,214 F: 77,241 HCC: 141 M: 71 F: 70	Mean age: 63.4	Wholegrains and dietary fiber		Wholegrains (T3 vs. T1): HR = 0.63; 95% CI: 0.41–0.96; *p* = 0.04 Total bran (T3): HR = 0.70; 95% CI: 0.46–1.07 Cereal fiber (tertile 3): HR = 0.68, 95% CI: 0.45–1.03; *p* = 0.06 Fiber from vegetables (T3): HR = 0.81, 95% CI: 0.54–1.21; *p* = 0.42 Fiber from fruits (T3): HR = 1.39, 95% CI: 0.88–2.21; *p* = 0.20
Li et al. (2014) [30] 1995–2011	NIH-AARP Diet and Health Study Prospective cohort study	United States	*n* = 494,942 M: 295,283 F: 199,659 HCC incident cases: 509	Mean age range = 50–71 years	Healthy Eating Index-2010 and Mediterranean Diet Score	FFQ	HEI-2010 (highest quintile) = HR, 0.72, 95% CI: 0.53–0.97; P trend = 0.03 aMED = HR, 0.62, 95% CI: 0.47–0.84; P trend = 0.0002
			NHS and HPFS cohort studies				
Ma et al. (2019) [32] 1976–32 year follow up 1986–32 year follow up	Nurses’ Health Study (NHS) and Health Professionals Follow-up Study (HPFS) Prospective cohort study		M (HPFS): 51,529 F (NHS): 121,700 HCC: 160	Mean age range M: 40–75 years Mean age range F: 30–55 years	Alternative Healthy Eating Index-2010, Alternate Mediterranean Diet and Dietary Approaches to Stop Hypertension		AHEI-2010 (highest tertile) = HR, 0.61 (95% CI: 0.39–0.95; P trend = 0.03) AMED; HR = 0.75; 95% CI: 0.49–1.15; P trend = 0.18)DASH; HR = 0.90; 95% CI: 0.59–1.36; P trend = 0.61)
Ma et al. (2019) [31] 1976–32 year follow up 1986–32 year follow up			M (HPFS): 51,529 F (NHS): 121,700 HCC: 163		Intake of total meats, processed red meat, unprocessed red meat, poultry, fish		Processed red meats (highest vs. lowest tertile intake levels) = 1.84 (95% CI: 1.16–2.92, P trend = 0.04) Total white meats (highest vs. lowest tertile intake levels) = 0.61 (CI: 0.40–0.91, P trend = 0.02) Unprocessed red meats = HR, 1.06 95% CI: 0.68–1.63, P trend = 0.85) Poultry = HR, 0.60, 95% CI: 0.40–0.90, P trend = 0.01)Fish = HR, 0.70, 95% CI: 0.47–1.05, P trend = 0.10)
Sui et al., (2019) [35] 1976–27.9 year follow up 1986–27.9-year follow-up			M (HPFS): 51,492 F (NHS): 88,783 HCC: 162	Mean age: M: 59.7 F: 67.8 Mean BMI: M: 25.4 F: 25.9 Diabetes: M: 6.4% F: 3.6% Mean age: 62.5 Mean BMI: 25.1 Diabetes: 5%	Nut consumption (tree nuts, walnuts, peanuts, peanut butter, etc.)		Total nut consumption (highest vs. lowest tertile intake, HR, 0.84; 95% CI, 0.56–1.26) Tree nut consumption = HR, 0.64, 95% CI: 0.43–0.95) NS association with peanuts (*p* = 0.90) walnuts (*p* = 0.19), peanut butter (*p* = 0.34)
Yang et al. (2020) [44] 1976–32 year follow up 1986–32 year follow up			*n* = 144,845 M (HPFS): 51,418 F (NHS): 93,427 HCC cases: 164		Dairy products (total, milk, butter, cheese and yoghurt)		Total dairy (highest vs. lowest tertile) = HR 1.85 (95% CI: 1.19–2.88; *p* = 0.009) High-fat dairy (HR = 1.81, 95% CI: 1.19–2.76; *p* = 0.008) Butter (HR = 1.58, 95% CI: 1.06–2.36; *p* = 0.04) NS association with yoghurt = HR, 0.72, 95% CI: 0.49–1.05; *p* = 0.26

Significant Effect (*p* < 0.05); No Effect (*p* > 0.05). Abbreviations: HR, Hazard Ratio; CI, Confidence Interval; OR, Odds Ratio; RR, Relative Risk; n, Population Size; M, male; F, female; PLC, primary liver cancer; HCC, hepatocellular cancer; FFQ, food frequency questionnaire; BMI, body mass index; HEI-2010, Healthy Eating Index-2010; AHEI-2010, Alternative Healthy Eating Index-2010; AMED, Alternate Mediterranean Diet; DASH, Dietary Approaches to Stop Hypertension; PUFA, polyunsaturated fatty acids; NHS, Nurses’ Health Study; HPFS, Health Professional Follow-up Study.

**Table 4:** Critical appraisal of the 30 studies with the use of the Quality Criteria Checklist.

Study	Relevance				Validity										Outcome
	1	2	3	4	1	2	3	4	5	6	7	8	9	10	
Bamia et al. (2015) [20]	NA	Y	Y	NA	Y	Y	NA	Y	NA	Y	Y	Y	Y	Y	+
Bamia et al. (2015) [21]	NA	Y	Y	NA	Y	Y	NA	Y	NA	Y	Y	Y	Y	Y	+
Bogumil et al. (2019) [22]	NA	Y	Y	NA	Y	Y	NA	Y	NA	Y	Y	Y	Y	Y	+
Chen et al. (2018) [23]	NA	Y	Y	NA	Y	Y	Y	Unclear	NA	Y	Y	Y	Y	Y	+
Duarte-Salles et al. (2015) [24]	NA	Y	Y	NA	Y	Y	NA	Y	NA	Y	Y	Y	Y	Y	+
Duarte-Salles et al. (2014) [25]	NA	Y	Y	NA	Y	Y	NA	Y	NA	Y	Y	Y	Y	Y	+
Fedirko et al. (2013) [26]	NA	Y	Y	NA	Y	Y	NA	Y	NA	Y	Y	Y	Y	Y	+
Freedman et al. (2010) [27]	NA	Y	Y	NA	Y	Y	NA	Y	NA	Y	Y	Y	Y	Y	+
Inoue et al. (2005) [28]	NA	Y	Y	NA	Y	Y	NA	Y	NA	Y	Y	Y	Y	Y	+
Johnson et al. (2011) [29]	NA	Y	Y	NA	Y	Y	NA	Y	NA	Y	Y	Y	Y	Y	+
Li et al. (2014) [30]	NA	Y	Y	NA	Y	Y	NA	Y	NA	NA	Y	Y	Y	Y	+
Ma et al. (2019) [31]	NA	Y	Y	NA	Y	Y	NA	Y	NA	NA	Y	Y	Y	Y	+
Ma et al. (2019) [32]	NA	Y	Y	NA	Y	Y	NA	Y	NA	NA	Y	Y	Y	Y	+
Polesel et al. (2007) [33]	NA	Y	Y	NA	Y	Y	Y	Y	N	Y	Y	Y	Y	Y	+
Rizk et al. (2019) [4]	NA	Y	Y	NA	Y	Y	Y	Y	N	Y	Y	Y	Y	Y	+
Setiawan et al. (2015) [34]	NA	Y	Y	NA	Y	Y	NA	Y	NA	Y	Y	Y	Y	Y	+
Sharp et al. (2005) [7]	NA	Y	Y	NA	Y	Y	Y	NA	N	Y	Y	Y	Y	Y	+
Stepien et al. (2016) [10]	NA	Y	Y	NA	Y	Y	NA	Y	N	Y	Y	Y	Y	Y	+
Sui et al. (2019) [35]	NA	Y	Y	NA	Y	Y	NA	Y	NA	NA	Y	Y	Y	Y	+
Koh et al. (2016) [36]	NA	Y	Y	NA	Y	Y	Y	Y	NA	NA	Y	Y	Y	Y	+
Kuper et al. (2000) [37]	NA	Y	Y	NA	Y	Y	Y	Y	NA	Y	Y	Y	Y	Y	+
Kurozawa et al. (2004) [38]	NA	Y	Y	NA	Y	Y	Y	Y	Unclear	NA	Y	Y	Y	Y	+
Lan et al. (2018) [39]	NA	Y	Y	NA	Y	Y	Y	Y	Unclear	NA	Y	Y	Y	Y	+
Montella et al. (2007) [40]	NA	Y	Y	NA	Y	Y	Y	Y	NA	NA	Y	Y	Y	Unclear	+
Tamura et al. (2018) [41]	NA	Y	Y	NA	Y	Y	Y	Y	NA	NA	Y	Y	Y	Y	+
Turati et al. (2014) [14]	NA	Y	Y	NA	Y	Y	Y	Y	NA	NA	Y	Y	Y	Y	+
Vogtmann et al. (2013) [42]	NA	Y	Y	NA	Y	Y	Y	Y	NA	NA	Y	Y	Y	Y	+
Yang et al. (2019) [43]	NA	Y	Y	NA	Y	Y	Unclear	N	NA	NA	Y	Y	Y	Y	+
Yang et al. (2020) [44]	NA	Y	Y	NA	Y	Y	NA	Y	NA	NA	Y	Y	Y	Y	+
Zhang et al. (2013) [45]	NA	Y	Y	NA	Y	Y	Y	Unclear	NA	NA	Y	Y	Y	Y	+

The Academy of Nutrition and Dietetics Evidence Analysis Library (EAL) and the Quality Criteria Checklist were used as the appraisal tools. NA, not applicable; Y, yes; N, no. Positive (+) = most of the answers to the validity questions are ‘Yes’ (including criteria 2, 3, 6, and at least one additional ‘Yes’). (Details of the questions were included in the Table 2).

## Abbreviations

HBV	hepatitis B virus
HCV	hepatitis C virus
HCC	hepatocellular carcinoma
IGF-1	insulin-like growth factor
MAFLD	metabolic-associated fatty liver disease
PRISMA	Preferred Reporting Items for Systematic Reviews and Meta-Analysis
PLC	primary liver cancer
PUFA	polyunsaturated fatty acids
